# Extreme local recycling of moisture via wetlands and forests in North-East Indian subcontinent: a Mini-Amazon

**DOI:** 10.1038/s41598-023-27577-5

**Published:** 2023-01-10

**Authors:** Akash Ganguly, Harsh Oza, Virendra Padhya, Amit Pandey, Swagatika Chakra, R. D. Deshpande

**Affiliations:** 1 Geosciences Division, Physical Research Laboratory, Navrangpura, Ahmedabad, 380009 India; 2 Indian Institute of Technology Gandhinagar, Gandhinagar, 382355 India

**Keywords:** Hydrology, Stable isotope analysis, Wetlands ecology, Climate change, Software

## Abstract

Moisture recycling in precipitation is an important hydrological process, accounting for ~ 67% globally. North-east India, home to the world's wettest place, boasts vast wetlands and forest-cover. Despite its proximity to the coast, we find locally recycled moisture to be the primary annual source of rainfall (~ 45%). During the pre-monsoon season, the enriched δ^18^O (~ − 0.7 ‰) and high d-excess (~ 14 ‰) are ascribed to enhanced transpiration, owing to atmospheric instability which causes Nor’westers. During the Monsoon season, oceanic flux provides increased surficial moisture, enabling deep-localised convection via evaporation. Significant localised recycling, even during the Monsoon season is estimated (~ 38%), with predominantly high d-excess in precipitation during latter half of the monsoon with increased moisture contribution from floods in Brahmaputra (high d-excess). The increasing δ^18^O and d-excess during the post-monsoon season is associated with progressively lesser rainout history and increased localized recycling (~ 67%). In light of the dwindling wetlands and forest-cover, our study highlights their indispensable role in governing regional hydro-meteorology and water availability.

## Introduction

The enormous evaporation (4.60 × 10^5^ bcm/year) from the oceans is the most important component of the global hydrological cycle. However, ~ 90% of the evaporated vapour precipitates back into the oceans, leaving only a small fraction (4.9 × 10^4^ bcm/year) of it reaching the continents. The continental precipitation received globally amounts to 1.20 × 10^5^ bcm/year, thus necessitating 8.1 × 10^4^ bcm/year (~ 67%) from continental recycling of moisture, in order to balance the global water budget^1^.

The distribution of global continental recycling is extremely inhomogeneous, with places like Amazon basin (high) and Australia (low) lying on both extremes of the spectrum^2^. Similarly, India having very large variability in precipitation pattern is also expected to have a large degree of spatial inhomogeneity in continental recycling. The Indian water budget dictates ~ 40% of continentally recycled moisture in precipitation^3^. North-East India, home to the wettest place on the planet (Mawsynram), is a major biodiversity hotspot^4^. This region experiences dynamic weather governed by complex orography, with the Himalayas to the north and the Shillong Plateau further south, coupled with presence of the largest (by volume) Indian river (Brahmaputra)^5^, as well as vast wetlands and forest cover^6,7^, akin to the Amazon. Thus, we expect a maximum (>> 40%) role of localized recycling in this region. The existing literature also identifies this region with maximum localized recycling in India. However, the maximum estimated contribution of recycled moisture from the existing studies is limited to ~ 25%^2,8,9^.

**Figure 1:**
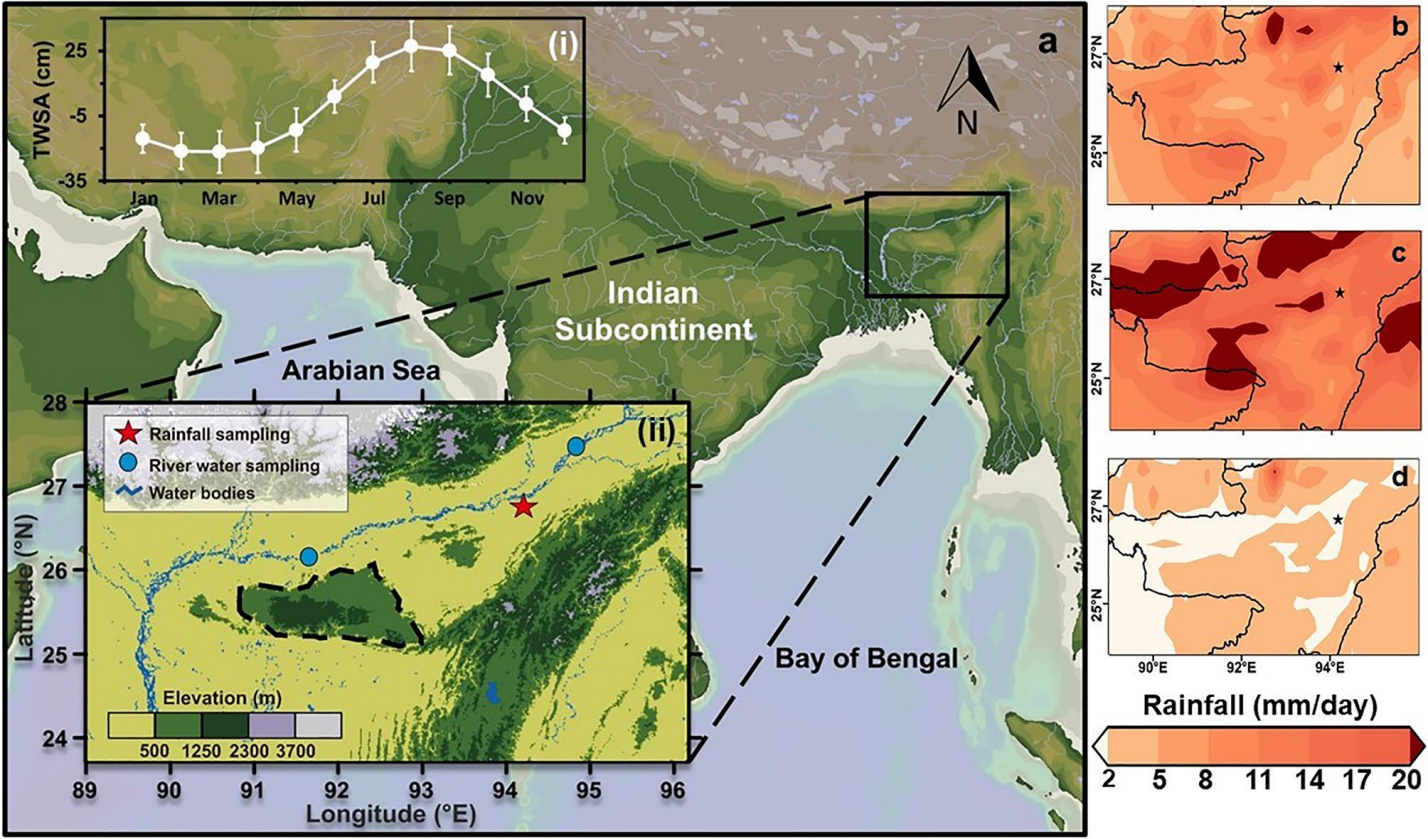
Study Area. (**a(i)**) GRACE TWSA seasonal variation during 2008–2013 indicating surface moisture availability in the region. The error bars represent standard deviation. (**a(ii)**) Map of North-East India depicting the study region. Jorhat, our precipitation sampling station is marked on the map (red star), along with location of major river sampling locations (blue circle). (**b**–**d**) The monthly climatology of rainfall amount for the pre-monsoon, monsoon, and post-monsoon season respectively in the region from 1979 to 2018 has been obtained using ERA 5 reanalysis dataset.

The average annual rainfall of NE India is ~ 2000 mm, and ~ 25% of it rains during the pre-monsoon (March–May) period^7,10,11^. The pre-monsoon heavy rainfall is associated with Nor’westers (atmospheric instabilities caused when warm and moist southwest winds are overrun by the cold and dry westerlies^12^). During the ISM (Indian Summer Monsoon, June–September) season, NE India receives ~ 1500 mm of rainfall. This hefty rainfall together with glacial melting during summers causes severe floods in Brahmaputra River^5,13^, and observed through the GRACE (Gravity Recovery and Climate Experiment) TWSA (Terrestrial water storage anomaly) seasonal fluctuations, indicating increased water availability^14^ (Fig. 1). These floods refill the water in low lying flood plains and widespread wetlands of the region.

The stable isotopes of oxygen(δ^18^O) and hydrogen(δ^2^H) in water are efficient tracers of hydrological processes, as their isotopic values vary predictably during physical processes such as evaporation, condensation, sublimation and deposition^15^. Several isotopic studies in the Amazon have already identified the significant role of the abundant wetlands and forest cover in recycling^16–18^. Declining forest cover and wetlands in the Amazon have been reported to affect the precipitation pattern remarkably^19,20^. Like the Amazon, several studies have also documented worrying trends of declining forest cover and wetlands along with studies suggesting the change in regional precipitation patterns for North-east India^21,22^. Previous studies have attempted to explain the variability of the ISM rainfall with the help of speleothem-based oxygen isotope reconstruction in the region coupled with use of ISOGSM2^23,24^. Such approaches however are hindered by coarse spatial resolution of ISOGSM2 model, sparse availability of speleothem records as well as complex orography and moisture dynamics. A continuous time-series measurement of stable water isotopes in rain as well as surface/river water can help shed light on the moisture transport pathways as well as identify major sources of moisture in the region. However, the isotopic studies in NE India are very limited^8,25,26^.

With this backdrop, the current study aims to decipher the role of Shillong Plateau and Brahmaputra in influencing the regional hydro-meteorology, and to estimate seasonal contribution of continentally recycled moisture in regional precipitation using stable isotopes (δ^18^O, δ^2^H), satellite-observations, reanalysis data as well as HYSPLIT (Hybrid Single-Particle Lagrangian Integrated Trajectory) model simulations.

## Results and discussion

### Pre-monsoon

**Figure 2:**
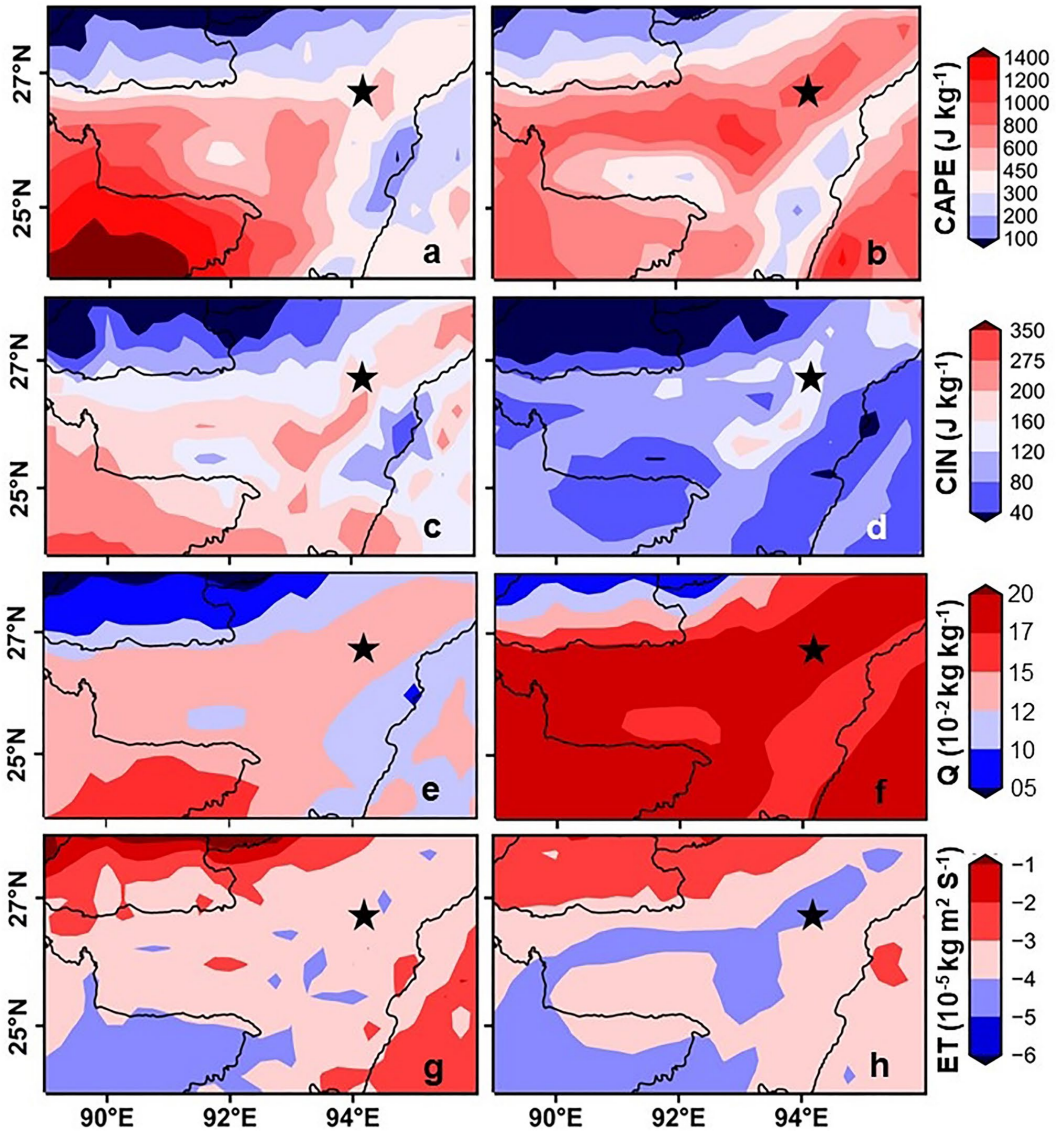
Meteorological evidence of localised moisture recycling. (**a**, **c**, **e**, **g**) The seasonal climatology of CAPE, CIN, Q and ET during Pre-monsoon (Mar–May) computed for the period 1979–2018 with the help of ERA 5 reanalysis dataset. (**b**, **d**, **f**, **h**) Similar plot but for the period of the ISM season (Jun–Sep).

During the pre-monsoon period, NE India receives a significant amount of rainfall (500 mm) owing to Nor’westers. Nor’westers carry moisture from the BOB further inland, therefore its effect on the regional hydro-meteorology is more pronounced south of the Shillong Plateau as observed from relatively high values of CAPE (Convective Available Potential Energy), Q (specific humidity at surface), ET (Evapotranspiration) flux (Fig. 2a,c,e,g) and is accompanied by frequent thunderstorms along with heavy rainfall^10,27^ when compared to the rest of India^28,29^. This contrasts with observations directly north of the Shillong Plateau. The pre-monsoonal rainfall received over this region renders the atmosphere moist and conditionally unstable, hence promoting convection^26,30^.

**Figure 3:**
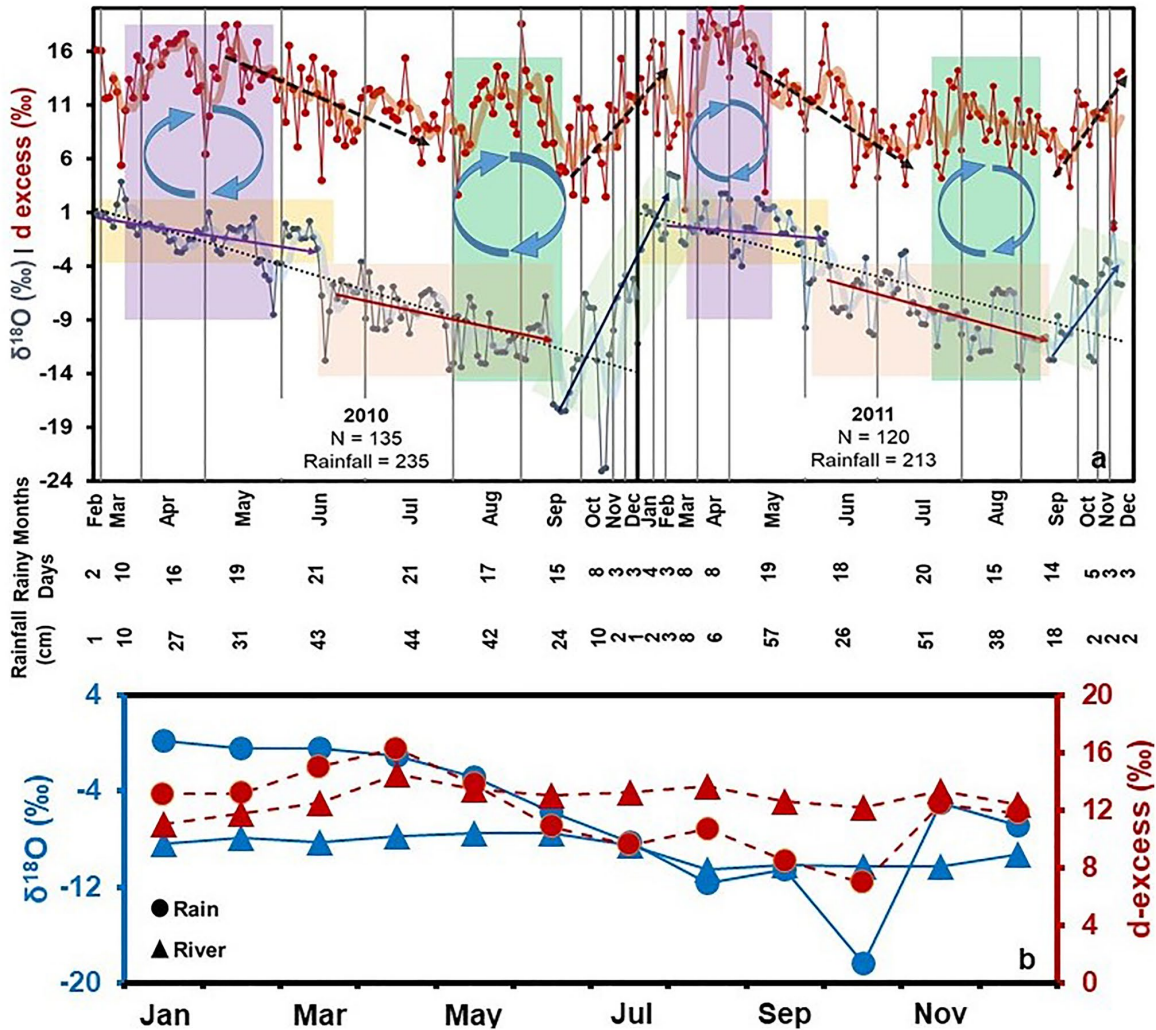
Isotopic trends in Precipitation and river water. (**a**) The daily isotope records of precipitation collected at Jorhat during 2010–2011. The enriched δ^18^O and high d-excess during Pre-monsoon (Mar–May) coupled with conspicuous slightly increasing trend of d-excess during latter half of the ISM (June–Sep) is indicative of hugely important role of locally recycled moisture contributing to precipitation. (**b**) The seasonal variation of δ^18^O and d-excess for precipitation (circle) and Brahmaputra (triangle) river water samples. Brahmaputra data shows overall high average d-excess and depleted δ^18^O.

During the Pre-monsoon season, we observe highly enriched values of δ^18^O (~ − 0.7‰) and high d-excess (~ 14‰) in daily precipitation samples collected from Jorhat, located to the north of the Shillong Plateau (Fig. 3a). Chakraborty et al.^8^ have tried to attribute higher values of δ^18^O in precipitation to the increased role of localized recycling dominated by transpiration. They have further explained this by suggesting uptake of progressively evaporated (isotopically enriched) soil moisture by the plants, so that the transpired vapour is isotopically enriched. While this argument can explain the enriched δ^18^O, it fails to explain the increased d-excess because evaporation decreases the d-excess of residual soil moisture^15,31–33^. This high d-excess can only be explained if the source d-excess values are significantly high.

**Figure 4:**
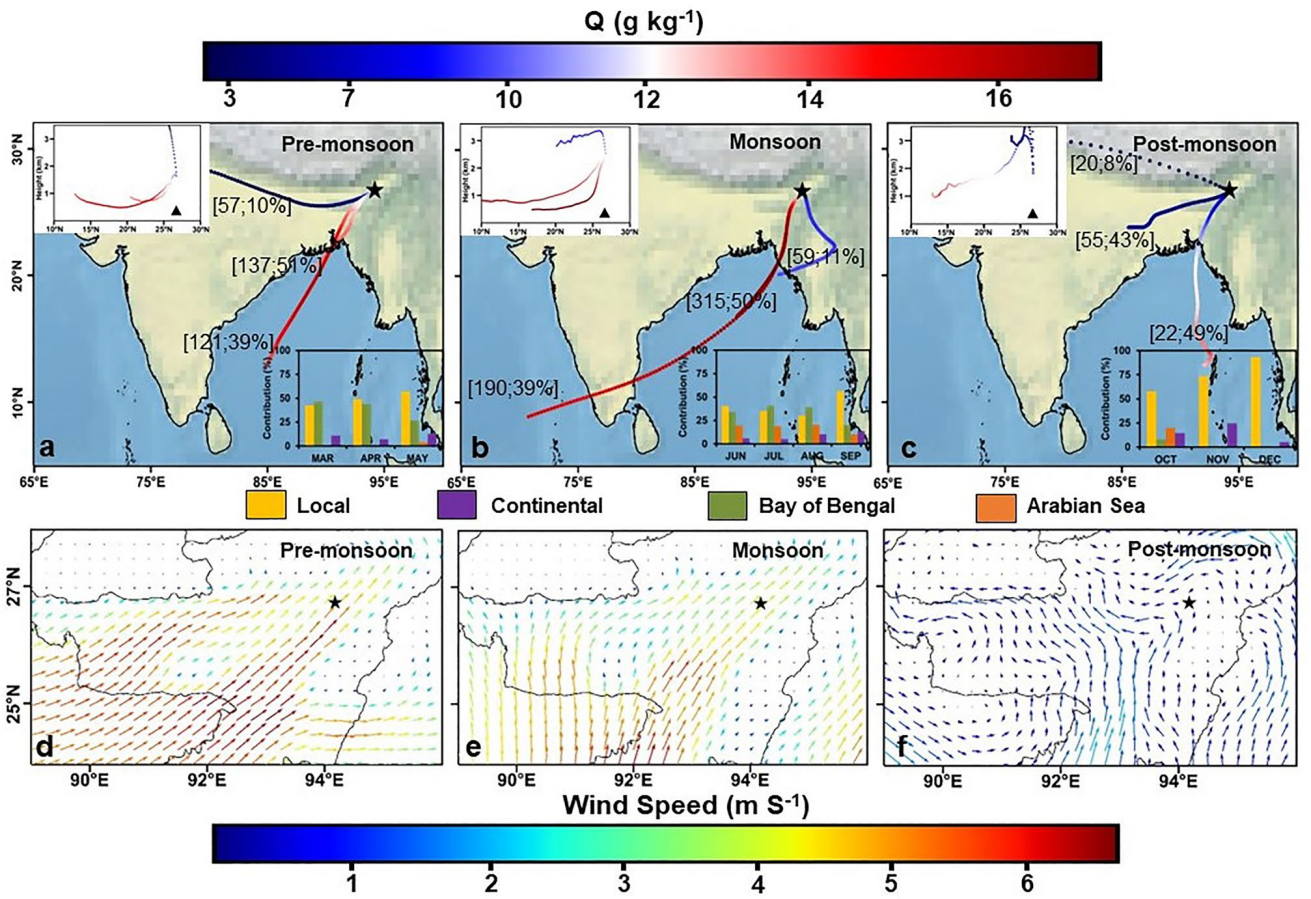
HYSPLIT moisture source allocation. (**a**–**c**) HYSPLIT clustered 120-h backward wind trajectories. The colormap at the top signifies specific humidity and moisture carried by the clustered mean trajectory. The numbers in bracket signify the frequency of trajectories in a cluster as well as the percentage of moisture transported along the respective clustered trajectory. The inset at top left represents the same plot but for height vs latitude variations. This highlights the role of Shillong Plateau as an orographic barrier in blocking oceanic winds. The inset at bottom right indicates monthly variation in moisture source contribution. (**d**–**f**) The monthly climatological wind maps derived at 850 hPa during 1979–2018. The wind maps indicate changing wind direction and strength during each season.

We hypothesise that the flooding in Brahmaputra [major source of high d-excess (~ 12.8‰)] during the previous years’ ISM period, recharges the surface as well as subsurface water, which in turn is transpired by the plants^5,13^. Our hypothesis is further substantiated by similar d-excess values in precipitation and river water (Fig. 2b) and higher localized recycling (~ 53%), estimated with the help of a simple empirical model based on HYSPLIT backward wind trajectories (Fig. 4a). In the HYSPLIT simulations, we have identified four major sources for the origin of vapour- Bay of Bengal, Arabian Sea, Local recycling, and continental which refers to the rest of recycled moisture but not of local origin (refer Section "Hysplit clustered trajectories" for more details).

The increasing trend in monthly estimated localized recycling (inset; bottom right Fig. 4 a) concurs with the increasing trend of evapo-transpiration given by Chakraborty et al.^8^.

The Shillong Plateau serves to demarcate this region into two distinct halves, with the enriched isotopic values in precipitation (δ^18^O ~ − 0.7‰) observed to the north, which is in contrast to the relatively depleted values reported by Breitenbach et al.^25^, Laskar et al.^34^, and Tanoue et al.^35^ (δ^18^O ~ − 2.4 ‰), to the south of the Plateau. The data reported in IAEA/WMO global network of isotopes in precipitation (GNIP) for the stations at Dhaka (2009–2018) and Sylhet (2009–2016), which are in Bangladesh to the south of the Shillong Plateau, also show similar relatively depleted trends during the pre-monsoon season.

Although, based on Rayleigh distillation, one would have expected a successive decreasing trend in both δ^18^O and d-excess with longer rainout history^15,32^. This suggests a more dominant role of isotopically depleted BOB moisture to the south of the plateau, whereas its role on the north is diminished due to the orographic barrier of Shillong Plateau. In the south of the plateau the enhanced values of CAPE, ET and Q indicate a greater role of localized recycling compared to the north (Fig. 2a,c,e,g). However, the huge influx of BOB moisture dilutes the isotopic footprint of recycled moisture in the south of plateau. On the other hand, these meteorological parameters indicate lesser role of localized recycling to the north, but the unavailability of significant BOB moisture gives room to the recycled moisture to express its isotopic signature in the precipitation.

The highest values of δ^18^O (~ 0.8‰) are observed during March along with lower d-excess (~ 11.7‰) compared to April–May (δ^18^O ~ − 1.2‰, d-excess ~ 14.7). This observation indicates significant evaporation from the falling raindrop due to high temperature and low rainfall^36^. The same can also be inferred from the lower value of LMWL (Local Meteoric Water Line) slope (6.96) for March, when compared to the GMWL (Global Meteoric Water Line)^37^. Towards the end of the pre-monsoon season, a systematic slight depletion in both δ^18^O and dexcess is observed. This can be ascribed to the small influx of isotopically depleted BOB moisture^25,38^circumventing the Shillong plateau, as seen from the wind patterns as well (Fig. 4d).

### Indian summer monsoon

North-east India receives maximum (~ 1500 mm) rainfall during the ISM season (Jun-Sep). ISM is set about with the northward migration of the ITCZ (Intertropical Convergence Zone), thus driving a huge influx of oceanic [BOB and AS (Arabian Sea)] moisture inland. Unlike Pre-monsoon, the regional hydro-meteorology to the north and south of the Shillong Plateau are analogous (Fig. 2b,d,f,h). The huge influx of oceanic moisture drives a sharp increasing trend of available surface water both to the north and south of the Plateau. This in turn results in lowering the BLH (Boundary Layer Height, Supplementary Fig. 1) and CIN (Convective Inhibition) and increase in ET, CAPE, and Q values (Fig. 2b,d,f,h).

Breitenbach et al.^25^ and Laskar et al.^34^ have reported an overall decreasing trend in δ^18^O at Mawlong and Hailakandi respectively, located immediately south of the Shillong Plateau. A similar trend is reported by Tanoue et al.^35^ at three locations in Bangladesh (Dhaka, Sylhet, Chittagong), which is further corroborated with GNIP data from Dhaka and Sylhet. We also observe a similar depleting trend at Jorhat in the first half of monsoon (June-July). Within the depleting trend, a baseline shift in δ^18^O can be observed in mid of June coinciding with the onset of the ISM, which is marked by a depletion in δ^18^O by ~ 4–5 ‰ (Fig. 3a) as well as an increase in the GRACE TWSA values. Following this baseline shift, there is a continuous trend of depletion in both δ^18^O and d-excess. This can be accounted for by the huge influx of oceanic moisture driven by northward migration of organized convection^35^, carrying isotopically depleted δ^18^O and lower d-excess. The oceanic contribution in precipitation estimated using HYSPLIT for June-July is ~ 57%. Even with the ISM and huge oceanic influx, the locally recycled contribution is significant (~ 37%). This is because unlike Pre-monsoon, the moisture laden winds brought by the oceanic influx approach the Shillong Plateau almost perpendicularly (Fig. 4b,e). This serves to limit the oceanic contribution to precipitation to the north of the Plateau and results in forced orographic ascent which is evident from Fig. 4b (inset, top left), with marked ascent of air parcel near 25°N coinciding with the start of the Plateau. Furthermore, owing to increased moisture availability and cloud cover, the transpiration rate is significantly reduced^26,39^, thus limiting the contribution of enriched transpired moisture to precipitation^8,40^. As a result, we do not observe an enriching trend in δ^18^O associated with significant moisture recycling.

Despite the homogeneity in hydro-meteorological parameters to the north and south of the Plateau, we observe a disparity in our observed d-excess trend when compared to the decreasing trend reported by Breitenbach et al.^25^ and Laskar et al.^34^ in the latter half of the ISM season (Aug–Sep). We would have expected a decreasing trend in d-excess accompanied by a depleting trend in δ^18^O at Jorhat on account of successive Rayleigh distillation process^15,32^. However, we observe a depleting trend in δ^18^O without any corresponding trend in d-excess (Fig. 3a). A similar conspicuous isotopic pattern akin to Jorhat is also observed at Tezpur, also located to the south of the Shillong Plateau^38,41^. One of the causal factors for the observed disparity could be the weakening of the ISM due to southward propagation of the ITCZ leading to lowering of oceanic influx. This is augmented by the orographic barrier posed by the Shillong Plateau, further hindering the oceanic contribution to the north of the Plateau^42^. Another causal mechanism is that in the latter half of the ISM, this region experiences frequent floods in Brahmaputra^5,13^, which is known to bring glacial fed water having depleted δ^18^O (~ − 10.3 ‰) and high d-excess (~ 13.1‰)^43^ (Fig. 3b). The increased surface water availability leads to favourable conditions promoting local recycling of moisture via evaporation^2^. The admixture of Brahmaputra moisture (high d-excess, low δ^18^O) with BOB influx (low d-excess, low δ^18^O) could explain the depleting trend in δ^18^O without any discernible accompanying trend in the d-excess. The increased proportion of locally recycled moisture is also corroborated with the higher recycled contribution (~ 39% local, ~ 11% continental), accompanied by a drop in oceanic contribution (from ~ 57% to ~ 50%), estimated using HYSPLIT (inset; bottom right Fig. 4b).

**Figure 5:**
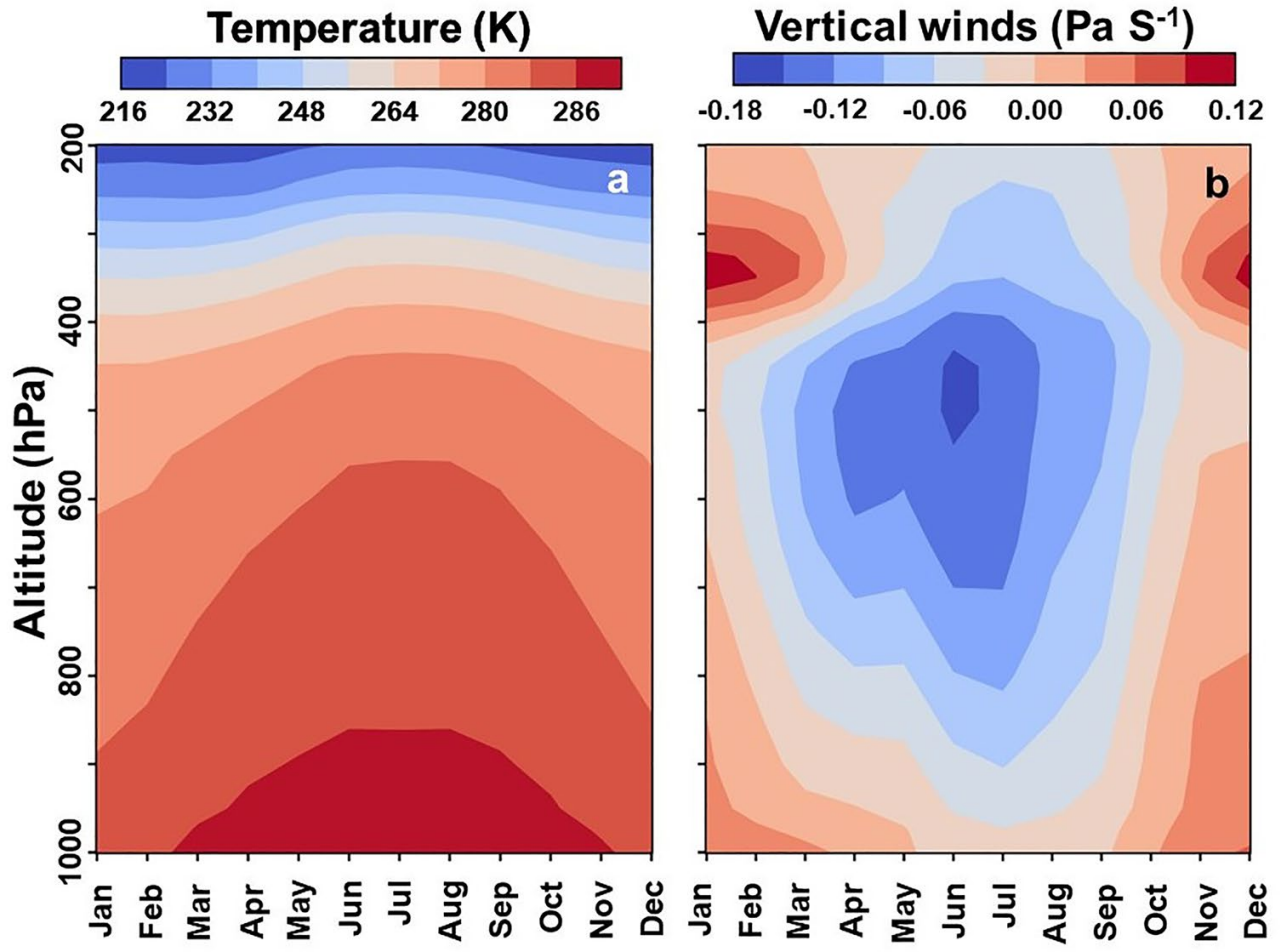
Deep Convective cells observed during the ISM season. The vertical profile for monthly air temperature and vertical wind velocity for the period from 1979 to 2018 highlighting formation of deeper convective cells during the ISM season. These deeper convective cells formed during ISM seem to be smaller in geographical extent than large scale organized convective cell, and localised as evident from variation in CAPE, CIN and ET values over a smaller geographical extent (See Fig. 2). The plots are generated for the rectangular grid ranging from 23.72°N–28°N, and 89°E–96.18°E.

From the foregoing, even during the ISM season, the locally recycled moisture plays an important role to the north of the Shillong Plateau. The overall estimated contribution from localized recycling in precipitation is ~ 38%. This result indicates a relatively higher proportion of localized recycling compared to the previous studies in this region^2,8,9,26^. The high proportion of recycling reported in our study is strongly backed up by the underlying regional hydro-meteorology. As with the increased surficial moisture during the ISM season, there is a significant lowering of viscosity in the boundary layer, hence lowering BLH^44,45^ (Supplementary Fig. 1). This is further accompanied by a reduction in the CIN and an increasing trend in spatial extent of higher CAPE values coinciding with the regions having high Q at the surface, which further gets accentuated post-floods. Also, from Fig. 5, we observe significant updraft beginning nearer to the surface and extending to higher altitudes, indicating deeper localized convective cells drawing moisture from the surface. A combination of all the above-mentioned factors together results in an environment conducive to stronger local recycling.

### Post-monsoon

North-east India receives very scanty rainfall during the Post-monsoon (Oct–Dec) due to the retreat of the ITCZ^46^, resulting in systematic decline of oceanic contribution along with weakening of monsoonal winds (Fig. 4f). The backward wind trajectory map also suggests that the distance traversed by the winds progressively reduces from Oct–Dec, thus drawing moisture from further inland. The estimated contribution of locally recycled moisture also shows an increasing trend from Oct (~ 58%)–Dec (~ 93%). We observe a progressively increasing trend in both δ^18^O and d-excess in precipitation (Fig. 3a) with shortened rainout history (Fig. 4c).

### Implications of high moisture recycling

Our study has brought to light the vital role of locally recycled moisture to precipitation throughout the year (~ 45%), at least to the north of the Shillong Plateau. During the Pre-monsoon season, we highlight the increased role of transpired moisture to precipitation, hence, signifying the importance of vegetation and forest cover. During the ISM season, influx of oceanic moisture and floods in Brahmaputra greatly increases the surficial water availability in low lying floodplains and wetlands, promoting enhanced recycling via evaporation.

Several studies have reported dwindling wetlands and forest cover due to overexploitation, urbanisation, siltation and deforestation^6,47,48^. This puts an increased stress on ecosystems and biodiversity as well as loss of livelihood and economy. Owing to climate change, a notable shift in precipitation pattern has been reported^49,50^. On top of this, our study highlights the vital role of forest cover and wetlands in the regional hydrology and precipitation patterns. Further anthropogenic activities such as rapid urbanization, building of dams or barrages could greatly endanger the regional hydrology and water availability.

## Methods

### Isotopic analysis

We collected daily rainwater samples at Jorhat (26.72°N, 94.18°E) situated in the state of Assam (marked in Fig. 1a(ii)), for the period 2010–11. We also collected samples from the Brahmaputra River during 2008–2015 with most samples collected from two sites Pandu (26.17°N, 91.67°E) and Dibrugarh (27.50° N, 94.84°E).

The oxygen and hydrogen isotopic analyses (δ^18^O and δD) were done by standard equilibration method in which water samples are equilibrated respectively with CO_2_ and H_2_. The equilibrated CO_2_ and H_2_ gases were analysed in isotope ratio mass spectrometer Delta V Plus in continuous flow mode using Gas bench II at Physical Research Laboratory (PRL), Ahmedabad, to measure the ^18^O/^16^O and D/H ratios to compute the δ^18^O and δD values^51^. The four secondary laboratory standards prepared in bulk and stored at PRL were analysed by Isotope Hydrology Laboratory at IAEA, Vienna on special request from IWIN National Program to obtain its authentic δ^18^O and δD values. Based on repeated analyses of multiple aliquots of these four secondary laboratory standards, the reproducibility of measurement was found to be better than 0.1 ‰ for δ^18^O and 1 ‰ for δD.

The oxygen and hydrogen isotopic composition are expressed in terms of abundance ratios of heavy to light isotopes (R = ^18^O/^16^O or D/H) and reported as δ in per mil (‰) notation [δ^18^O or δD = (R_sample_/R_std_ − 1) × 1000]. R_std_ is the ratio in the Vienna Standard Mean Ocean Water (VSMOW). Another derived parameter used in this study is d-excess (= δD − 8 × δ^18^O), defined by Dansgaard, 1964 to study kinetic effects associated with evaporation of water. The temporal variation in the δ^18^O, δD and d-excess rain, in conjunction with meteorological parameters has been used to discern various processes and factors such as vapor source, rainout history, localized recycling and role of Shillong Plateau^36,52–54^.

### Reanalysis data

Both long-term as well as daily hydro-meteorological parameters were derived from ERA 5 dataset (https://www.ecmwf.int/en/forecasts/datasets/reanalysis-datasets/era5). In order to monitor the behaviour of CAPE, CIN, ET, and other meteorological parameters over a broader scale, we have prepared monthly climatology for the study area (23.72°N–28°N, and 89°E–96.18°E) from 1979 to 2018 with resolution of 0.25° × 0.25°. The long-term climatological records are used to validate whether the understanding of possible causal rain-forming mechanisms obtained with the help of isotopic records is consistent with the underlying hydro-meteorology of the region.

It is necessary to compute the height at which maximum convergence and condensation in clouds occurs and its variation with season in order to accurately represent the rain forming pathways with the help of HYSPLIT backward wind trajectories. In order to resolve this, we estimate the pressure and geopotential height above msl (mean sea level) for which the CRWC (Cloud Specific Rain Water Content) peaks and assume it to best represent the approximate cloud condensation height. This exercise is repeated for those days during 2010–2011 when we observe rainfall at Jorhat. Monthly mean pressure for CRWC maxima is computed from the daily estimates. HYSPLIT backward wind trajectories are then generated with their respective cloud condensation height for each month.

### HYSPLIT backward wind trajectories

In the present study wind, trajectory analyses were done using Hybrid Single Particle Lagrangian Integrated Trajectory (HYSPLIT) Model from https://www.ready.noaa.gov/HYSPLIT.php^55^. The backward trajectories were generated with Jorhat (26.72°N, 94.18°E) as the starting location for the HYSPLIT run. Ensembles of four 120 h back trajectories starting at every 6-h interval (00:00, 06:00, 12:00, 18:00) were obtained for every daily rain event. Thus, for each daily rain event, four trajectories were monitored to understand moisture source location.

#### Hysplit moisture source estimation

For the daily rain samples, we obtain the pressure/geopotential height in a 1° × 1° grid centred around Jorhat for which the CRWC is maximum. Monthly estimates for pressure/altitude at which CRWC maxima occurs is obtained by taking the mean over rainy days for the particular month. This estimate is used to initialise the height at which the backward trajectories converge over Jorhat. Specific humidity is used as a proxy for moisture in air parcels, with decrease attributed to precipitation and increase mapped to moisture pickup from the source region based on Sodemann et al.^56^. We identify four distinct sources of moisture (a) Bay of Bengal (b) Arabian Sea (c) Local (d) Continental. In order to ascertain the component labelled as ‘Local’, we consider the region covering North-eastern part of Indian subcontinent including part of north-western Myanmar. The component labelled as ‘Continental’ accounts for the rest of the recycled moisture that is not derived locally. We observe that the moisture derived from Bay of Bengal, Arabian Sea, and Local components account for ~ 91% of the total contribution and are sufficient to identify the major moisture regions.

The relative contributions from the four sources are calculated at every hourly interval for each trajectory. The four trajectories considered for any given day have different percentage contributions from each source. Hence, their contributions are amount weighted wrt the final specific humidity reported at Jorhat for the respective trajectories. In order to obtain monthly/annual values, we amount weight the daily estimates with the amount of daily rainfall. The detailed methodology is described in Oza et al.^57^.

#### Hysplit clustered trajectories

HYSPLIT backward wind trajectories provide an efficient tool in identifying major moisture source pathways and source locations. Despite this, when studying over hundreds of backward wind trajectories together, representing them graphically, in a concise and coherent fashion could be a challenge. Clustering provides an efficient way to obtain grouping of similar trajectories, hence providing a clear and concise graphical representation of different moisture transport pathways.

The conventional HYSPLIT trajectory clustering technique attempts to group trajectories based on similarity between their respective endpoints with the help of a spatial variance method^55–58^. Hence, we end up neglecting the altitude-based information which would be otherwise invaluable in identifying convection as well as understanding the role of orographic barriers. Furthermore, the spatial variance method only includes information about the trajectory endpoints, thus discounting any variations that might have occurred further along the trajectory pathway.

Hence, we have invoked an improved clustering algorithm incorporating latitude, longitude as well as altitude at every 6 hourly intervals along the trajectories’ journey. This approach can capture the role played by orographic barriers as well as highlight localised convection which cannot be conveyed from just a 2-dimensional representation.

For each trajectory we consider the latitude, longitude, altitude above msl at every 6-h interval along the 120 h of its journey to reduce the volume of data. Furthermore, Principal Component Analysis (PCA) is performed to further reduce the dimensionality of the dataset. The first eight principal components are considered since they preserve > 95% of the explained variance. Kmeans algorithm is used along with ‘means++’ initialisation for the purpose of clustering^59^. Elbow method is used to determine the ideal number of clusters for different seasons. Specific humidity is plotted with the help of a colour bar along with the trajectory to map the moisture pickup regions.

### Gravity recovery and climate experiment (GRACE)

GRACE level 3 gridded data was used to observe TWSA with 2008–2013 as the baseline wrt which the anomalies are computed since this covers the period of both our as well as some past studies^14^. The optional scaling factors/ gridded gain factors have been multiplied to the original product to make it comparable to model data such as GLDAS. The TWSA data was used to infer the period during which we observe a sudden increase in moisture availability in the region, indicative of onset of the ISM as well as advent of floods.

## Supplementary Information

Supplementary Figure 1.

## Data Availability

The authors declare that the reanalysis data can be obtained from https://www.ecmwf.int/en/forecasts/datasets/reanalysis-datasets/era5. The HYSPLIT backward wind trajectories can be generated from https://www.ready.noaa.gov/HYSPLIT.php. The GRACE TWSA data can be found at https://grace.jpl.nasa.gov/data/get-data/. The GNIP data used in the study can be accessed at http://www-naweb.iaea.org/napc/ih/IHS_resources_gnip.html. The stable water isotopic data as well as code used to run the models are available from the corresponding author upon reasonable request.
