# Supplementary material for: Extreme local recycling of moisture via wetlands and forests in North-East Indian subcontinent: a Mini-Amazon
Source: Sci Rep. 2023 Jan 10;13:521. doi: 10.1038/s41598-023-27577-5 (PMC9831987; doi:10.1038/s41598-023-27577-5)
Supplement: Supplementary file 1 — Supplementary Figure 1. [file 41598_2023_27577_MOESM1_ESM.docx]

**Extreme Local Recycling of Moisture via wetlands and forests in North-East Indian Subcontinent - a Mini-Amazon**

Akash Ganguly*^1 ,2^, Harsh Oza^1^, Virendra Padhya^1^, Amit Pandey^1^, Swagatika Chakra^1^, R.D. Deshpande^1^

^1^Geosciences Division, Physical Research Laboratory, Navrangpura, Ahmedabad, 380009, India

^2^Indian Institute of Technology Gandhinagar, Gandhinagar, 382355, India


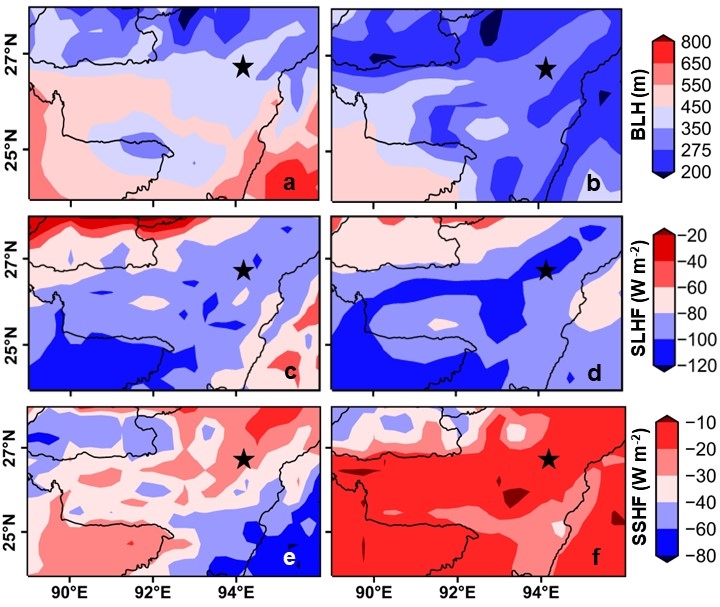
 **Fig. S1: Meteorological Evidence of Localised Moisture Recycling**

**a, c, e** The seasonal climatology of BLH, SLHF and SSHF during Pre-monsoon (Mar-May) computed for the period 1979-2018 with the help of ERA 5 reanalysis dataset. b, d, f Similar plot but for the period of ISM (Jun-Sep)
